# Orthopedic manifestations as presenting symptoms in children with neuroblastoma: a retrospective case series and clinical review

**DOI:** 10.1186/s13018-025-06610-5

**Published:** 2026-02-12

**Authors:** Siddarth Kamath, Kumar Amerendra Singh, Hitesh H. Shah

**Affiliations:** Department of Paediatric Orthopedics, Kasturba Medical College, Manipal Academy of Higher Education (MAHE), Manipal, Karnataka India

**Keywords:** Neuroblastoma, Orthopedic manifestations, Musculoskeletal symptoms, Pediatric oncology

## Abstract

**Introduction:**

Neuroblastoma is the second most common childhood malignancy. Only a minority of children with metastatic disease present initially to orthopedic surgeons, despite musculoskeletal complaints such as back pain, limb pain, limp, extremity swelling, or findings mimicking osteomyelitis. These vague and nonspecific presentations increase the risk of delayed diagnosis.

**Aims:**

To study orthopedic manifestations that present as the initial presenting symptoms of neuroblastoma in children and to characterize their clinical, radiological, and laboratory profiles.

**Materials and methods:**

Forty-six consecutive patients with neuroblastoma were retrospectively evaluated. Medical records were reviewed, with particular attention given to the presence of orthopedic manifestations preceding the diagnosis of neuroblastoma. The children who were presented primarily to the orthopedics department were identified. The details of musculoskeletal symptoms and radiological and laboratory investigations were analyzed.

**Results:**

Seven children (20%) presented initially with orthopedic complaints. Three patients had spinal involvement, including paraplegia from hydromyelia or vertebral metastasis with lytic–sclerotic lesions. Three children presented with persistent hip pain and limp and were initially diagnosed with osteomyelitis before biopsy confirmed neuroblastoma. One child presented with nontraumatic forearm swelling, initially presumed as osteomyelitis, with radiographs showing lysis of the ulnar metaphysis. A biopsy was used to establish the diagnosis. Six children had severe anemia with elevated ESR and CRP, and three had markedly elevated LDH levels.

**Conclusion:**

Approximately one-fifth of children with neuroblastoma initially present to orthopedic surgeons. Persistent or atypical musculoskeletal complaints—especially hip pain or back pain accompanied by anemia, high ESR, or high CRP—should prompt the consideration of neuroblastoma and early histopathological evaluation to avoid diagnostic delays.

## Introduction

Neuroblastoma is the most common extracranial solid tumor in childhood [1, 2]. It typically arises from primordial neural crest cells of the sympathetic nervous system [1, 15]. Most commonly, the primary tumor is located in the adrenal glands, the sympathetic chain or the lung [1, 15]. Up to 60–70% of children suffering from neuroblastoma present with metastatic disease with high affinity toward the bone and bone marrow [2, 4]. Consequently, orthopedic symptoms ranging from bone pain, limp, and pathological fractures to spinal cord compression are common [3].

The correct diagnosis of neuroblastoma may be delayed because of the evaluation of common local and generalized systemic differentials of a limping child [1]. Local bone diseases such as osteomyelitis; bone tumors such as Ewing sarcoma or osteosarcoma; occult tuberculosis; septic arthritis; Perthes disease; and systemic diseases such as rheumatic disease, leukemia and sickle cell disease pose significant diagnostic dilemmas [1, 11].

25% of children suffering from neuroblastoma present primarily to orthopedic surgeons with symptoms that mimic these common differential diagnoses [3]. Some studies have shown a delay of 15 days to 5 months in establishing a diagnosis of neuroblastoma due to vague presentations [6, 7].

For treatment to be effective, it is paramount to diagnose these rare tumors as early as possible [4]. In the literature reviewed, these orthopedic manifestations are not well described.

The objectives of this study were as follows:

1. To evaluate the orthopedic manifestations that serve as initial presenting symptoms in children later diagnosed with neuroblastoma.
2. To evaluate the clinical, radiological, and laboratory characteristics associated with thesepresentations.
3. Evaluating diagnostic pathways and delays to identify opportunities for earlier recognition.

## Materials and methods

### Study design

A retrospective case series compliant with the STROBE guidelines was conducted in the Department of Pediatric Orthopedics at a tertiary care hospital between January 2006 and December 2024.

## Data collection

The records of all consecutive children aged less than or equal to 18 years at diagnosis with biopsy-confirmed neuroblastoma were included in the study. Children with incomplete medical or imaging data or with other musculoskeletal oncological conditions were excluded from the study.

## Data collection

**Fig. 1:**
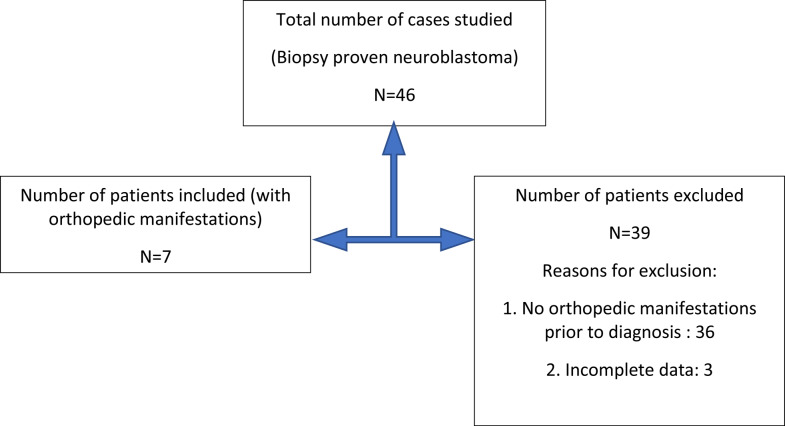
Flowchart showing patient inclusion for the study

Data were extracted from the hospital records using a structured proforma Fig. 1. The following variables were studied:

*Clinical presentations* Symptoms in the child, such as pain, limp, bone or joint swelling, back pain or spinal compression.

*Radiological investigations* X-rays, bone scan reports, and CT or MRI scans that revealed skeletal involvement.

*The laboratory findings* included the erythrocyte sedimentation rate (ESR), hemoglobin levels, platelet counts, serum lactate dehydrogenase (LDH) levels and blood peripheral smears.

LDH and blood peripheral smears were performed, as elevated LDH or abnormal smears suggest marrow infiltration or alternative hematologic malignancies with atypical clinical presentations.

*Initial diagnosis/source of referral* whether seen initially by the Pediatric Orthopedic Department or other departments.

*Time to diagnosis* the time interval between the presentation of symptoms and the establishment of a diagnosis of neuroblastoma by biopsy of the involved site or the bone marrow.

### Data analysis

Descriptive statistics were used to summarize the demographic and clinical data. Categorical variables are reported as frequencies and percentages. Continuous variables are expressed as the means Table 1.

Comparisons were made between patients who presented with limb involvement and those who presented with spine involvement using the chi-square test. A *p* value of < 0.05 was considered to indicate statistical significance. Data analysis was performed using SPSS for Windows Table 2.

## Results

### Demographic data

A total of 46 consecutive children with biopsy-proven neuroblastoma were included in the study. Seven children fulfilled the inclusion criteria and presented primarily to the orthopedic surgeon.

**Table 1:** Summarizing demographic data

Sex (*N* = 7)	Male = 4	Female, *N* = 3
Mean age at presentation (Mean ± SD) (years)	4.3 ± 2	
Mean duration of follow up (Mean ± SD) (months)	20 ± 6	

### Prevalence of orthopedic manifestations

**Table 2:** Various orthopedic manifestations

Orthopedic manifestation	Number of cases
Pain in the bone or joint	4 (3 children: Pain in the hip joint, 1 child with painful swelling of forearm)
Limp or refusal to walk	3
Back pain or spinal symptoms	3
Pathological fracture	1 (distal ulna)

Painful limp due to effects on the hip joint was the most common presenting symptom.

### Radiological features (Figs. 1 and 2)

**Fig. 2:**
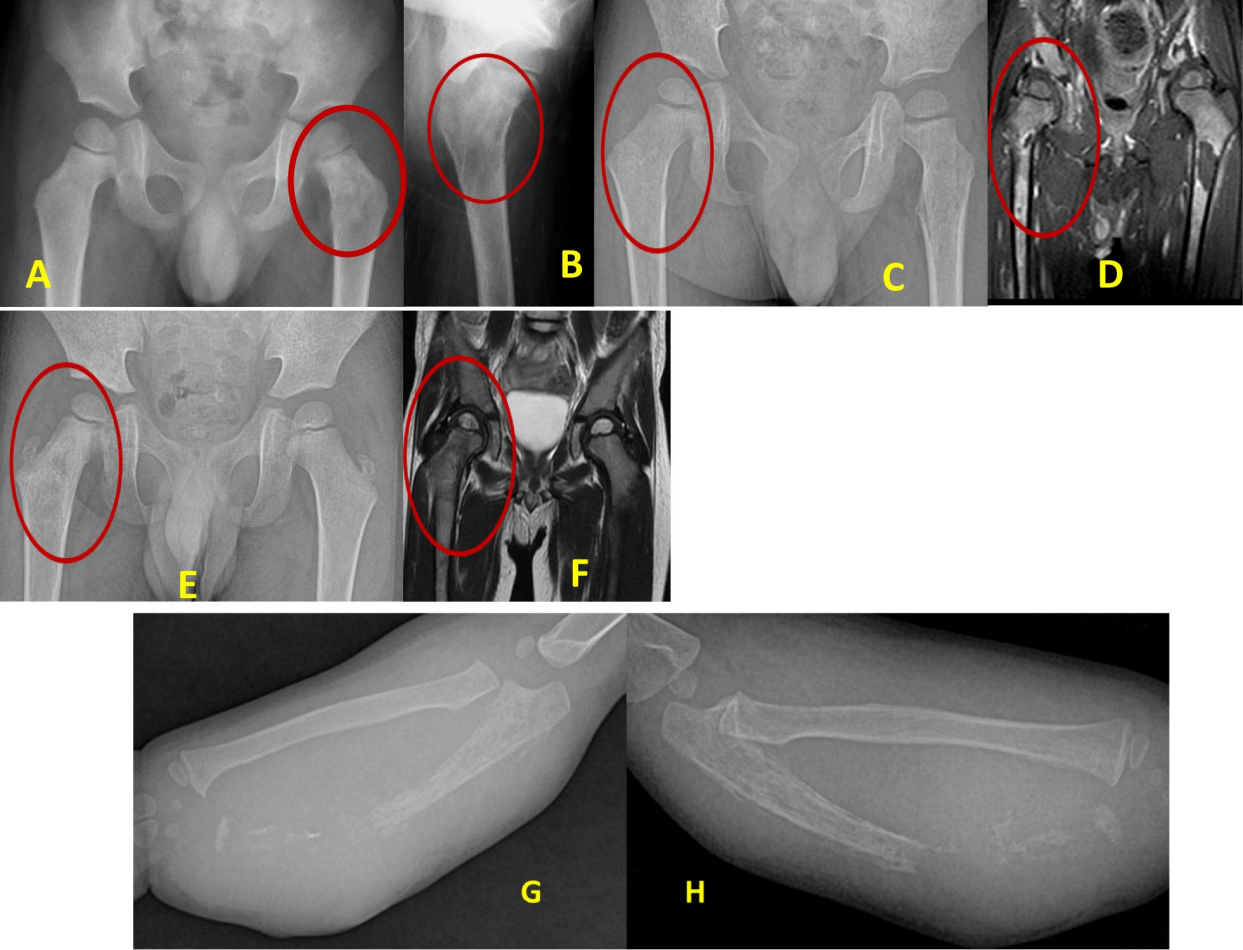
**A**–**F**. Osteolytic lesions marked with red circles. Figure **A** and **B**—Showing child 1, who presented with left hip pain and painful limp. X-rays revealed lytic lesions over the proximal femur. The child had anemia and elevated ESR and CRP levels. Child underwent biopsy of the lesion, revealing the diagnosis of neuroblastoma. Figures **C** and **D** show child 2, and **E** and **F** show child 3, who presented with right hip pain and painful limps. X-rays revealed a subtle lytic lesion over the proximal femur. MRI was performed and revealed osteomyelitis. The child had severe anemia with elevated ESR, CRP and LDH levels. A biopsy and histopathological examination revealed a diagnosis of neuroblastoma. **G** and **H** – Child 4, who presented with painful swelling over the forearm. X-rays revealed a lytic lesion over the distal ulna with pathological fracture. A differential diagnosis of osteomyelitis was suspected. The child had anemia and elevated ESR and CRP levels. A biopsy was performed, which revealed a diagnosis of neuroblastoma

**Fig. 3:**
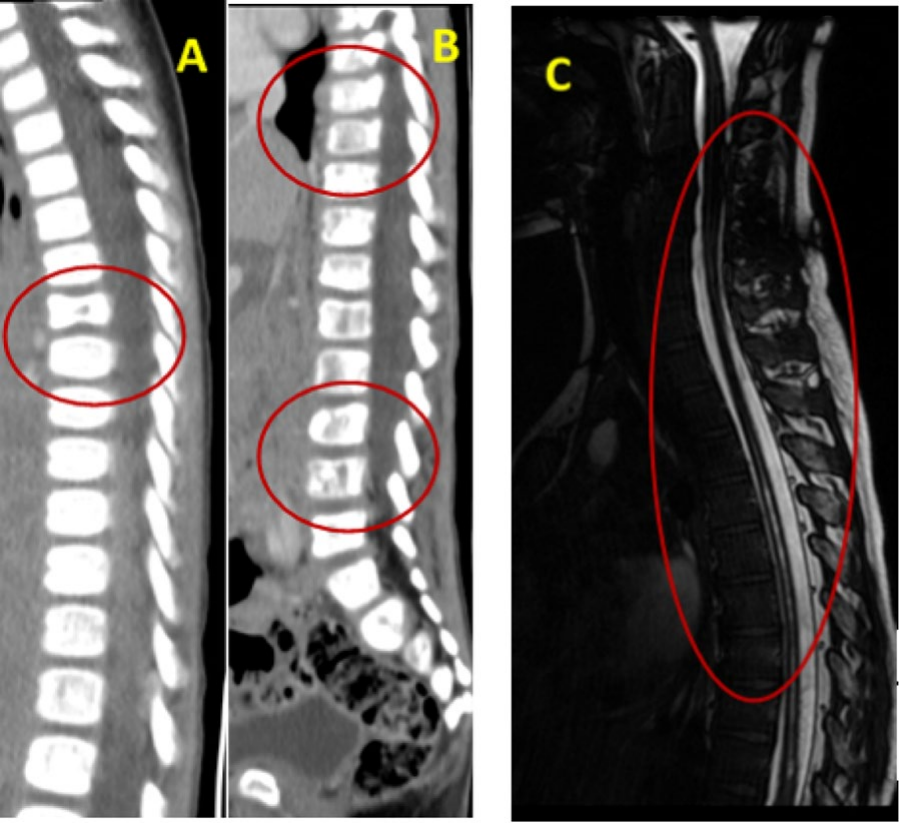
(**A**–**C**). Osteolytic lesions marked with red circles. Figure **A** shows child 5, Figure **B** shows child 6, who presented with symptoms of spinal cord compression and paraplegia. CT scans revealed extensive metastatic lesions over the vertebral body. These lesions were lytic lesions with a surrounding zone of sclerosis. Bone marrow biopsy revealed a diagnosis of neuroblastoma.Figure **C**–Child 7 presented with infantile neuroblastoma with extensive vertebral metastasis and hydromelia. MRI revealed lesions and cord involvement. Bone marrow biopsy revealed a diagnosis of neuroblastoma

**Fig. 4:**
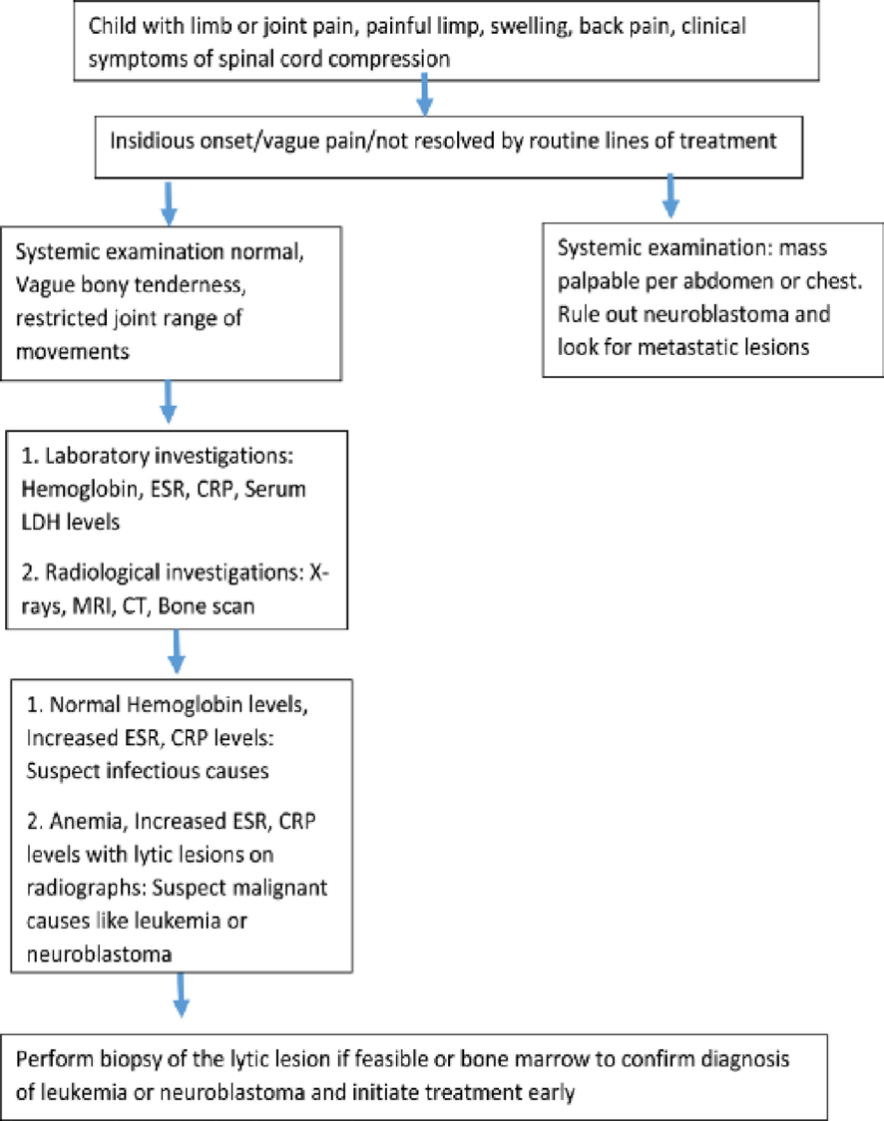
Diagnostic flowchart

Skeletal involvement on imaging was observed in all 7 children Fig. 3 and 4.

*Lytic bone lesions (X-rays)* Three children had lytic lesions of the proximal femur that mimicked osteomyelitis. One child had a lytic lesion on the distal ulna with a pathological fracture resembling osteomyelitis.

*Spinal lesions with cord compression (CT or MRI)* Three children had lytic lesions of the vertebral bone with surrounding zones of sclerosis.

#### Laboratory investigations

**Table 3:** Showing laboratory parameters

Parameter	Number of cases
Elevated ESR levels	6 (Mean ± SD = 94 ± 10)
Elevated CRP levels	6 (Mean ± SD = 85 ± 8)
Anemia	7 (Mean ± SD = 7.1 ± 1.3)
Elevated platelet counts	3
Elevated LDH levels	3

All the children who presented with orthopedic manifestations were anemic Table 3.

**Table 4:** Literature review of various studies on the musculoskeletal manifestations of neuroblastoma

References	*N*	Study type	Musculoskeletal manifestation	Laboratory investigations	Radiological investigations	Remarks	Limitations of the study
Busfield et al. [1]	6	Retrospective case series	Pain in joint or limb pain, Limp, Bone pain resembling osteomyelitis, Backache, Paraplegia	Increased ESR levels	Osteolytic bone lesions in the metaphysis, may be bilateral, could resemble onion skin appearance	–	Only ESR levels and Xray radiographs were studied. Other radiological or laboratory parameters were not evaluated
Ashton et al. [12]	20	Retrospective case series	Hip pain, Nonspecific limping, Limb weakness, Back pain	Low Hemoglobin, Elevated ESR levels	Perform bone marrow aspiration to confirm diagnosis	Ultrasound examination and hip aspiration to rule out infection	Suggested bone marrow aspiration only once ultrasound or hp aspiration results were equivocal. This could add to the diagnostic delay
Wong et al. [7]	1	Case report	Hip and Back pain	Increased ESR and LDH levels, Anemia	Perform bone scan in suspicious cases	–	Small sample size
Mohan et al. [5]	1	Case report	Irritable hip	Low Hemoglobin	Consider early MRI in vague presentations	–	Suggested MRI in suspicious lesions. However, MRI findings maybe inconclusive leading to delay in diagnosis
Thapa et al. [6]	2	Letter to editor	Multiple joint pains mimicking juvenile rheumatoid arthritis	Low hemoglobin, High ESR, LDH levels	Bone scan to look for marrow infiltration	–	Small sample size
Natrajan et al. [13]	1	Case report	Irritable hip	Severe anemia and presence of blasts on peripheral smear	–	–	Small sample size
Chu et al. [9]	2	Retrospective case series	Multiple joint pains mimicking juvenile rheumatoidarthritis	---	Abdominal examination and ultrasound evaluation in suspicious cases	–	Described only the imaging features of metastatic neuroblastoma
Parmer et al. [3]	4	Retrospective case series	Irritable hip	Anemia, High ESR	Targeted screening of abdomen with ultrasound along with hip ultrasound in suspicious cases	Follow with MRI if abdominal screening is negative	Some cases of neuroblastoma cold be extra abdominal. Stepwise diagnostic pathways could lead to delayed biopsy and confirmatory diagnosis
Amin et al. [8]	2	Retrospective case series	Irritable hip	Laboratory investigations are not specific	Suggested MRI	To keep patients with non specific hip pain in close follow up for upto 3 months	Deferring a biopsy could lead to diagnostic delays
DaSilva et al. [4]	1	Case report	Irritable hip	Anemia, High ESR, LDH	Bone scan to diagnose marrow infiltration in suspicious cases	–	Small sample size
Abousamra et al. [14]	2	Retrospective case series	Hip pain, painful limp	--	–	–	Small sample size
Current study	7	Retrospective case series	Irritable hip, Forearm swelling, Spinal metastasis, Paraplegia	Anemia, Increased ESR, CRP, Platelet and LDH levels	CT, MRI are not specific for diagnosis	Early consideration of histopathological examination of suspicious lesions or bone marrow	Treatment provided and long term follow-up is not discussed

N, Number of cases

#### Initial clinical misdiagnosis

Among the 7 children with orthopedic symptoms, 4 were initially misdiagnosed with osteomyelitis. Three children had proximal femur involvement, and 1 child had involvement of the forearm Table 4.

#### Time to diagnosis

The time from the onset of orthopedic symptoms to diagnosis was as follows:

- Limb involvement group: 4.5 weeks (SD 3.5–6).
- Spine involvement group: 2 weeks (SD 1.0–3.0).

This difference was statistically significant (*p* = 0.01).

#### Referral patterns

Four children with limb involvement were first seen by the pediatric orthopedic department, 3 children with spine involvement were first seen by the pediatric department, and referrals were sought from the pediatric orthopedic department.

## Discussion

This study presents one of the larger cohorts examining orthopedic manifestations as initial presentations of neuroblastoma, demonstrating that approximately one-fifth of children manifested musculoskeletal symptoms before diagnosis. Although this proportion is clinically significant, it likely reflects referral patterns to a tertiary orthopedic center and should be generalized cautiously. Nevertheless, these findings reinforce the importance of maintaining diagnostic vigilance when evaluating persistent or unexplained musculoskeletal complaints in children.

Bone or joint pain and limping were the most frequent presenting symptoms, which is consistent with prior studies reporting high rates of skeletal involvement in disseminated disease [3–6]. However, these symptoms remain nonspecific and overlap with several benign pediatric orthopedic conditions. Back pain and signs of spinal cord compression are more suggestive of serious pathology but typically represent advanced disease, limiting their utility for early detection. Previous studies often emphasize these features as diagnostic clues, but such signs usually appear only after significant tumor progression and may not improve timely diagnosis.

Misdiagnosis of osteomyelitis, which was encountered in four patients in this study, remains a well-documented challenge. Wong et al. and Busfield et al. [1, 7] reported similar diagnostic delays due to overlapping clinical and laboratory features between infection and malignancy. The average diagnostic delay of 4.5 weeks in our cohort was shorter than that in earlier reports; however, this difference may reflect increased institutional awareness rather than inherent improvements in distinguishing early disease. Children with spinal involvement are diagnosed more rapidly because of the urgency of neurological symptoms, again highlighting that dramatic presentations expedite diagnosis, whereas subtle symptoms often lead to delays.

Previous authors have recommended adjunct investigations—MRI, hip aspiration, bone marrow studies, scintigraphy, and abdominal ultrasound—when the clinical findings are atypical [3, 5, 7, 12]. However, much of this guidance stems from small series or case reports, and their diagnostic performance has not been systematically evaluated. Parmar et al. [3] suggested a screening ultrasound examination of the abdomen in children with atypical limp. However, neuroblastoma is known to involve extra-abdominal sites, which could be missed with an abdominal ultrasound and cause further delays in diagnosis. Our findings demonstrate that initial imaging, including MRI, frequently fails to confidently identify malignancy, highlighting the limitations of current modalities when used in isolation. The reliance on biopsy for definitive diagnosis is consistent with previous literature but also illustrates that delays may occur when biopsy is deferred in favor of stepwise testing for more common conditions.

The identification of an atypical metastatic site in the distal forearm further challenges the classical pattern described by Wong et al. and Chu et al. [7, 9], who emphasized axial and proximal appendicular involvement. Such atypical presentations complicate diagnostic reasoning and underscore the need for a low threshold to investigate lytic lesions that do not fit expected clinical patterns. In our cohort, most lesions were osteolytic with surrounding sclerosis, a finding that may mimic infection or healing of bone and contribute to misinterpretation. Although osteolysis has been widely described [2, 4], the variabilityof accompanying reactive changes is underrecognized in the literature and may hinder timely diagnosis.

The role of orthopedic surgeons as early identifiers of malignancy is widely acknowledged, yet prior literature may overstate the practicality of this expectation. Orthopedic clinics frequently encounter nonspecific pain and limp, and without structured evaluation pathways, early malignancy can be easily overlooked. Our findings support the need for standardized protocols for escalating evaluation—particularly when pain persists, imaging is inconclusive, or laboratory abnormalities coexist. Early multidisciplinary involvement may help mitigate diagnostic delays.

Overall, this study highlights both the importance and the limitations of orthopedic presentations as early indicators of neuroblastoma. While musculoskeletal symptoms should prompt consideration of malignancy in atypical cases, their low specificity, variable imaging features, and overlapping differential diagnoses continue to pose significant challenges. Future research should focus on identifying more reliable clinical or imaging markers and evaluating diagnostic algorithms that may facilitate earlier recognition of malignancies in pediatric orthopedic practice.

### Strengths

This study is one of the largest single-center studies to focus on the orthopedic manifestations of neuroblastoma. The clinical, radiological and laboratory features were characterized in detail. It includes diagnostic timelines, delays and common misdiagnostic patterns of this condition.

### Limitations

This was a single-center retrospective study. The sample size, although representative, may limit generalizability. The treatment details and long-term outcomes were not described in this study.

## Conclusion

The results of our study show that a child with neuroblastoma often presents primarily to orthopedic surgeons (20%). Children with atypical hip pain, back pain with severe anemia and elevated ESR and CRP levels should alert the treating orthopedic surgeon to suspect the differential diagnosis as neuroblastoma and consider histopathological confirmation to reduce delays in treatment.

## Data Availability

The datasets used and/or analyzed during the current study are available from the corresponding author upon reasonable request.
